# Ferroelectric capacitors and field-effect transistors as in-memory computing elements for machine learning workloads

**DOI:** 10.1038/s41598-024-59298-8

**Published:** 2024-04-24

**Authors:** Eunseon Yu, Gaurav Kumar K, Utkarsh Saxena, Kaushik Roy

**Affiliations:** School of Electrical and Computer Engineering, Purdue University, West Lafayette, IN 47907 USA

**Keywords:** Electrical and electronic engineering, Computational science

## Abstract

This study discusses the feasibility of Ferroelectric Capacitors (FeCaps) and Ferroelectric Field-Effect Transistors (FeFETs) as In-Memory Computing (IMC) elements to accelerate machine learning (ML) workloads. We conducted an exploration of device fabrication and proposed system-algorithm co-design to boost performance. A novel FeCap device, incorporating an interfacial layer (IL) and $\text {Hf}_{0.5}\text {Zr}_{0.5}\text {O}_2$ (HZO), ensures a reduction in operating voltage and enhances HZO scaling while being compatible with CMOS circuits. The IL also enriches ferroelectricity and retention properties. When integrated into crossbar arrays, FeCaps and FeFETs demonstrate their effectiveness as IMC components, eliminating sneak paths and enabling selector-less operation, leading to notable improvements in energy efficiency and area utilization. However, it is worth noting that limited capacitance ratios in FeCaps introduced errors in multiply-and-accumulate (MAC) computations. The proposed co-design approach helps in mitigating these errors and achieves high accuracy in classifying the CIFAR-10 dataset, elevating it from a baseline of 10% to 81.7%. FeFETs in crossbars, with a higher on-off ratio, outperform FeCaps, and our proposed charge-based sensing scheme achieved at least an order of magnitude reduction in power consumption, compared to prevalent current-based methods.

## Introduction

**Figure 1:**
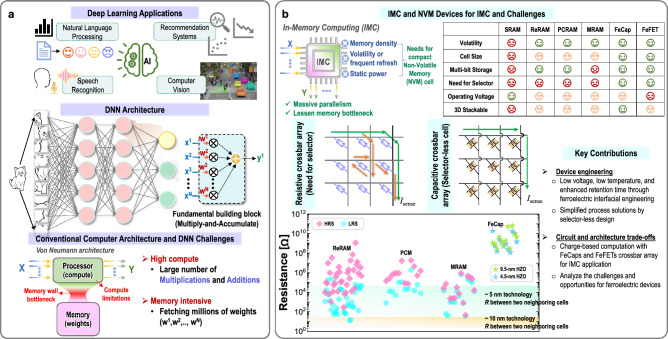
Deep-learning (DL) applications, associated challenges, and the need for in-memory computing (IMC) with Non-Volatile Memory (NVM) devices. (**a**) Holistic view of DL applications, the architecture of a fully connected neural network, and the challenges that allow IMC to complement. (**b**) IMC and NVMs aimed at this purpose, featuring the characteristics of crossbar architecture characteristics based on memristor devices and capacitive crossbar array for its counterpart. The resistance plot shows the values of the Low Resistance State (LRS) and High Resistance State (HRS) for various NVMs, and the internal resistances of FeCaps obtained from our experimental data (at different HZO thicknesses of 4.5 nm and 9.5 nm). When the width of crossbar wire is scaled from 10 to 5 nm^13^, there is a notable increase in its resistance. This increase brings it into a range comparable to the LRS of ReRAMs, PCMs, and MRAMs, yet maintaining distinction from the internal resistances of FeCaps. Our key contributions are highlighted.

The ubiquity of smart Internet of Things (IoT) devices, projected to surpass 29 billion by 2030^1^, commands a transformative influence on diverse aspects of contemporary life. Empowered by sophisticated machine learning (ML) capabilities, these devices are progressively being used in various applications, optimizing functionalities from real-time analytics to complex decision-making processes. A pressing challenge in this evolution lies in effectively managing the formidable computational demands intrinsic to ML workloads. Standard von Neumann-based hardware architectures have limitations in navigating these contemporary exigencies, chiefly manifested as the “memory-wall” or the “von Neumann bottleneck^2^”. This bottleneck (Fig. 1), marked by a significant speed gap between processors and memory, necessitates a pivot towards more innovative and responsive architectural paradigms.

In response, In-Memory Computing (IMC)^3^ has emerged as a promising architectural solution where memory devices are organized within a crossbar array^4^. This arrangement improves computational efficiency by enabling the parallel in-situ execution of essential neural network operations, particularly Matrix-Vector Multiplications (MVMs) and General Matrix Multiplications (GEMMs). Traditional CMOS memories, including Static Random Access Memory (SRAM) and Dynamic RAM (DRAM), do offer IMC solutions but come with their notable drawbacks. These include substantial static leakage and scalability issues, frequent refreshing, and the complexity of peripheral circuitry. These factors ultimately hamper their overall effectiveness in managing complex ML workloads, leading to increased latency and energy consumption.

Emerging Non-Volatile Memories (NVMs), like Resistive RAM (ReRAM)^5,6^, Phase-Change Memory (PCM)^7^, and Magnetoresistive RAM (MRAM)^8^, offer promising solutions against the limitations of conventional CMOS-based memories. Their reduced size and enhanced data retention capabilities make them favorable for IMC. For instance, ReRAMs can have a memory footprint approximately $3-5\times$ smaller than SRAMs and $1.5\times$ smaller than DRAMs, for similar technology nodes^9,10^. It’s worth noting that these ratios are calculated considering standard 2D layout geometries. Utilizing emerging 3D geometries in layout can significantly reduce the ReRAM cell area to $\le 4F^2$, providing $\ge 20\times$ area benefit compared to standard SRAMs^11^. However, the application of these NVMs in IMC presents complexities such as sneak path effects and crossbar parasitics (Fig. 1), challenging their practical utility. To overcome these challenges in NVMs, a large selector device is incorporated for each memory cell. However, this inclusion raises concerns about scalability, energy overhead, and process complexity, potentially offsetting the intrinsic benefits of NVMs’ compactness. Thus, it is imperative to develop selector-less memory cells to harness their true potential in IMC crossbars.

Ferroelectric crossbars, which consist of Ferroelectric Capacitors (FeCaps) or Ferroelectric Field Effect Transistors (FeFETs), have emerged as a compelling alternative. Notable attributes, including minimal leakage currents for enhanced energy efficiency and the absence of selector devices, along with their innate high internal resistance, foster compactness, and support larger crossbars. They are also compatible for integration with CMOS technology^12^.

FeCaps require a relatively lower operating voltage than FeFETs and have a compact two-terminal design, which allows for high memory density and a streamlined fabrication process. They embody the essence of traditional capacitors, mitigating DC current and reducing static power consumption, obviating the need for selector devices. In addition to the aforementioned benefits, FeFETs offer a high on-off ratio exceeding $10^4$. However, the integration of FeFETs and FeCaps in IMC comes with its own challenges. Despite these advantages, challenges arise when scaling the area of FeCap and FeFET devices due to the polycrystalline nature of the ferroelectric material, such as $\text {Hf}_{0.5}\text {Zr}_{0.5}\text {O}_{2}$ (HZO). This can hinder the performance^14^ and reliability^15^ of the devices, necessitating careful consideration in design^16^ and sensing periphery^17^. Additionally, for FeCaps, their inherent low ratio of high-capacitance state (HCS) to low-capacitance state (LCS) ($C_{ratio}$) leads to computational errors, an aspect that has been somewhat neglected in previous research^18–20^. On the other hand, FeFETs require high programming voltages and suffer from poor endurance performance.

This article discusses ferroelectric memory-based IMC in three phases: the development of low operational voltage devices, the design and analysis of crossbar arrays, and the exploration of impending challenges and associated trade-offs. Finally, we introduce a device-system co-design and architecture solutions to address these challenges.

In the pursuit of energy-efficient IMC units, a critical consideration is the reduction of the operating voltage for these devices. Typically, HZO-based FeCaps incorporate a 10-nm-thick layer which facilitates operation at voltages of around 2 V or higher. Our work places particular emphasis on the development of low-voltage ferroelectric devices, leading to 1.2 V operation, achieved through interfacial layer (IL) engineering and a thin HZO layer. Furthermore, the inclusion of IL not only improves ferroelectricity, but also enhances retention performance. The fabrication of proposed FeFET focuses on obtaining a sufficient memory window, demonstrating a 1-V memory window with conventional 10-nm HZO layer. The measured results of these fabricated devices are fitted to a physics-based model to characterize their behavior for performing crossbar array analyses, particularly for essential operations like MVMs, crucial for various ML workloads.

We identified inherent limitations of FeCaps as IMC elements, incorporating errors in computations, primarily stemming from their low $C_{ratio}$. To address these challenges, we propose a device-circuit-algorithm co-design solution. This approach takes into account the impact of a small $C_{ratio}$ during the neural network training phase, thereby extracting acceptable performance in FeCap-based IMC crossbars at inference. Our proposed methodology achieves an accuracy of 81.7% on the CIFAR-10 dataset using Resnet-20, compared to a mere 10.0% baseline accuracy in the absence of any pre-training. Moreover, we propose a modified crossbar architecture utilizing FeCaps that eliminates the need for pre-training, albeit with an additional cost in terms of area and power consumption. Furthermore, we introduce a charge-based inference scheme in FeFET crossbars, enhancing energy and area efficiency. This is achieved by eliminating power-hungry transimpedance amplifiers (TIA) and bulky load capacitors, resulting in over an order of magnitude lower power consumption. Our comparative analysis highlights the promising attributes of ferroelectric solutions, advancing our understanding of their applicability in the ever-evolving landscape of memory technologies and IMCs.

The rest of the article is organized as follows: the **Results** section presents the measurement data obtained from the fabricated ferroelectric devices and discusses the fitting of the simulation model to the experimental data. We also showcase the practical application of FeCaps and FeFETs in crossbar arrays for IMC, before delving into operational principles, associated challenges, and potential opportunities. Moving forward, the **Discussion** section throws light into the findings and insights on employing FeFETs and FeCaps as IMC elements in crossbars. The **Methods** section depicts the fabrication process for FeCaps and FeFETs, along with device characterization, before discussing simulation and training methodology.

## Results

### Ferroelectric devices as IMC elements

Recent research on ferroelectric devices have focused on their applications within the IMC framework. While there exist some complementary features between FeCaps and FeFETs, both devices boast extremely high internal resistance values as their most advantageous attributes as shown in Fig. 1b. These characteristics collectively enable the creation of a selector-less cell. We examine both FeCaps and FeFETs in the context of IMC applications.

**Figure 2:**
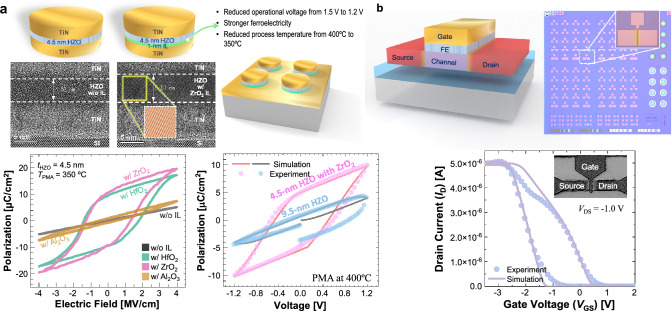
Schematics and performances of our fabricated FeCaps and FeFET. (**a**) High-resolution transmission electron microscopy (HR-TEM) images of FeCaps having a 4.5-nm HZO layer without an IL (left) and with 1-nm $\text {ZrO}_{2}$ IL (right). Polarization-electric field plots of the FeCaps having different IL materials along with 4.5-nm HZO (annealing temperature was 350 $^{\circ }$C). Without IL, the HZO layer remained as paraelectric material under the given annealing temperature. Simulation model fitting results with 9.5-nm and 4.5-nm HZO thicknesses devices. (**b**) Optical microscope image of our fabricated die. Fitted FeFET transfer curve obtained from the simulation model compared with the experimental data (inset: Scanning Electron Microscope (SEM) image).

#### Device characteristics and memory operation of ferroelectric devices

To substantiate the promise of ferroelectric devices, we fabricated FeCap and FeFET devices. These devices incorporated HZO as ferroelectric material. In the fabrication of FeCap, we considered the reduction of operational voltage and process temperature, along with enhancements in reliability^21^. Thinning the HZO layer can be a potent strategy for reducing operational voltage by leveraging the consistent coercive field of HZO material in various thicknesses. However, this exponentially increases the thermal budget required for ferroelectricity activation, which presents considerable challenges^22^. Extending the Back-End-of-Line (BEoL) process temperature undermines the reliability and performance of integrated CMOS peripherals, which are crucial for optimizing the overall area efficiency through 3-dimensional (3-D) stacking. To that effect, there are previous approaches such as employing the plasma-enhanced ALD (PE-ALD) method^23^, employing a material with a low thermal expansion coefficient^24,25^, and implementing a surface treatment technique^26^.

Our approach was tailored to balance these scaling considerations, simply leveraging a 1-nm IL using diverse materials such as $\text {HfO}_{2}$, $\text {ZrO}_{2}$, and $\text {Al}_{2}\text {O}_{3}$ in conjunction with a 4.5-nm or 9.5-nm HZO layer. The use of an IL demonstrated elevated ferroelectric performance, reduced the operational voltage, and reduced process temperature to 350 $^{\circ }$C (Fig. 2a)^21^. Notably, $\text {HfO}_{2}$ and $\text {ZrO}_{2}$, outperformed $\text {Al}_{2}\text {O}_{3}$, owing to their structural affinity with HZO, lower crystallization temperatures, and greater disparities in their thermal coefficients with HZO and/or electrode materials (particularly, $\text {ZrO}_{2}$ IL emerged as the leading candidate). These advancements represent significant improvements in FeCap technologies: (1) addressing the limitation imposed by the BEoL thermal budget of 400 $^{\circ }$C, given that an annealing temperature higher than 450$^{\circ }$C is necessary to secure sufficient ferroelectricity when the thickness of HZO is reduced below 4.8 nm^22^ and 5.6 nm^27^; (2) scaled the operational voltage from the baseline of 1.5 V to 1.2 V (typical 2 V or above observed in 10-nm HZO FeCap devices^22,28^ attributed to the coercive field of $\ge 1.0$ MV/cm); (3) improved the retention performance (Fig. S1).

In the fabrication of our FeFET, securing enough memory window and low leakage current were our foremost objectives. Our fabricated FeFET demonstrated a 1-V memory window with a 10-nm HZO layer, as shown in Fig. 2b. The on-off current ratio was $4.9\times 10^6$ (Fig. S2). While the FeFET need not be exclusively *p*-type, we chose to employ our in-house fabricated *p*-type FeFET device. The experimental data from both FeCaps and FeFETs were fitted to the Preisach model for crossbar array simulation^29^. Figures 2a,b and S2 showcase the results of the model fitting, highlighting the correlation between the experimental data and the simulation model for their respective devices.

**Figure 3:**
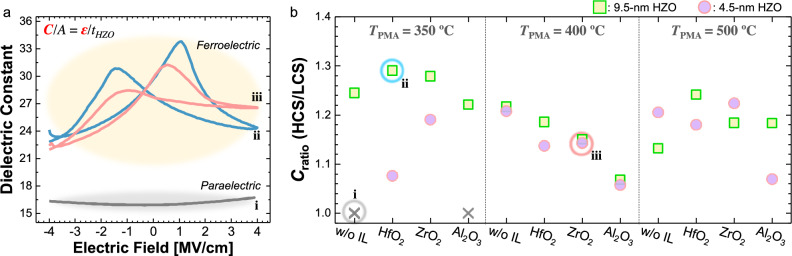
FeCap capacitance characteristic. (**a**) Butterfly-shape dielectric constant vs. electric field curves from three different FeCap devices. Corresponding devices are denoted as i, ii, and iii in **b**. (**b**) Maximum observed capacitance ratio (*C*$_{ratio}$) across FeCap devices.

The capacitances of FeCaps vary with electric field, and this facilitates the unique feature of selector-less cells in MVM operations. This capacitive crossbar array offers an advantage over resistive crossbar arrays, being more energy efficient by eliminating static power consumption. Based on the capacitance (*C*) equation ($C/A = \epsilon /t_{HZO}$), the dielectric constant ($\epsilon$) is the only variable since the device area (*A*) and the thickness of HZO ($t_{HZO}$) are the physically fixed values. Figure 3a exhibits dielectric constant values of three FeCaps, back-calculated from our capacitance measurement (Fig. S3). Ferroelectric devices have butterfly-shaped distinctive patterns arised from the change of its atomic structure in response to electric fields, an exclusive characteristic of the ferroelectric film. On the contrary, a paraelectric device showed a relatively consistent dielectric constant regardless of external electric fields.

Although an ideal butterfly-shaped capacitance-electric field *(E*) curve is symmetrical, practical considerations such as trapped charges, defects, and oxygen vacancies introduce observed asymmetry and shift the cross-point of the positive and negative direction electric field sweeps at non-zero *E* (Fig. 3a). This asymmetrical *C*-*E* curve finds valuable application in MVM, facilitated by the charge equation *Q* = *CV*. After programming pulses, FeCap devices are read at varying capacitance values (*C*), with the charge output (*Q*) determined by the input voltage (*V*). Figure 3b depicts the maximum capacitance ratios observed in our fabricated devices, which encompassed a variety of IL materials, HZO thicknesses, and process temperatures. The highest C$_{ratio}$s (= HCS/LCS) of 1.2 and 1.29 were observed for 4.5-nm and 9.5-nm HZO samples, respectively. In other words, the differences between the maximum and minimum dielectric constants were $5.83 \times \epsilon _{0}$ and $7.04 \times \epsilon _{0}$, respectively. Here, $\epsilon _{0}$ is the vacuum permittivity. For our simulation of the capacitive crossbar array, we used a FeCap device featuring a $\text {ZrO}_{2}$ IL with a 4.5 nm HZO layer, demonstrating a low-voltage operation of 1.2 V.

**Figure 4:**
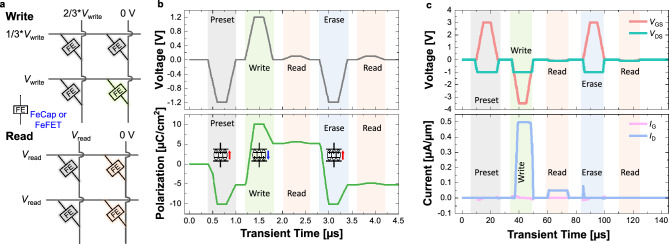
Memory operation and signalling schemes: (**a**) Write and read operation schemes in crossbar array. Timing diagram for (**b**) FeCap and (**c**) FeFET. Programming the FeCap requires an operating voltage of 1.2 V, whereas the FeFET programs at 3 V. The change in the polarization state controls the value of capacitance in FeCap and the value of conductance in FeFET.

Figure 4a shows write and read operations with voltage applications. Figure 4b,c illustrate the memory operation of a single FeCap and FeFET, respectively. Initially, a preset voltage is applied to align the polarization direction. Subsequently, a programming (write 1) pulse of 1.2 V and an erase (write 0) pulse of $-1.2$ V are applied, followed by a read operation at 0.1 V for each. In the case of FeCap, the use of a low read voltage of 0.1 V, way below the coercive voltage (higher than 0.7 V), ensures a non-destructive read operation^28,30^. This non-destructive read was confirmed by the unchanged polarization values of post-read operation (Fig. 4b).

FeFETs offer a significantly low leakage current with a high on-off ratio due to the superior switching characteristics of the transistors. The ferroelectric layer as the gate oxide of the FeFET encodes data within its polarization state. This, in turn, modulates the threshold voltage of the FeFET, effectively controlling the conductance of its channel (Fig. 4c). For FeFET operation, the programming employs a gate voltage (*V*$_{GS}$) of -3 V and the erase uses +3 V of *V*$_{GS}$, with a drain voltage (*V*$_{DS}$) of $-1.0$ V. During read, *V*$_{GS}$ = *V*$_{DS}$ = -0.1 V is used, ensuring a highly non-destructive read condition for the current polarization status of the FeFET. Based on the aforementioned operation conditions, we performed array simulations that will be explained in the following section.

**Figure 5:**
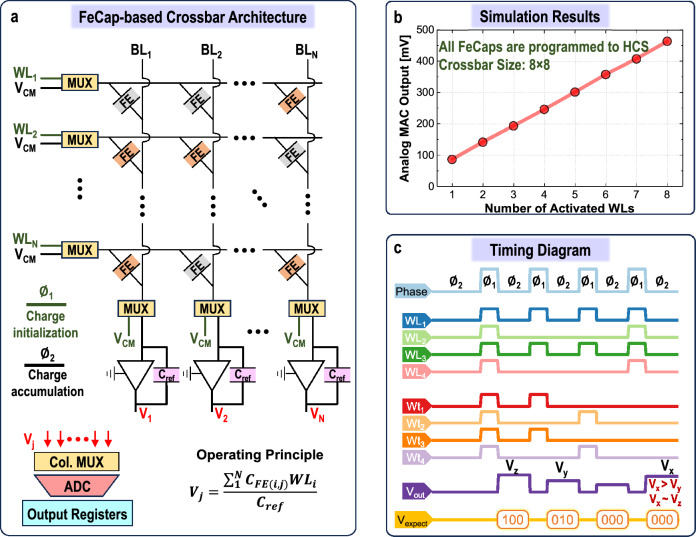
(**a**) FeCap-based IMC crossbar architecture for MVM computations. (**b**) Simulation results depicting the accumulated analog MVM (or MAC) output for $8 \times 8$ array, where FeCaps programmed to HCS and a number of WL (input) activations are varied. (**c**) Timing diagram for a $4\times 4$ array to show the operation of crossbars, illustrating the different scenarios that lead to erroneous calculations, which result from the inherent low HCS/LCS ratio.

### Crossbar array analyses

#### FeCap-based crossbar arrays for IMC

Based on the proposed FeCap devices, this work harnesses the inherent ferroelectric properties to encode information via capacitance states. Programming a low-capacitance state (LCS) or a high-capacitance state (HCS), as shown in Fig. 3a, is achieved by applying electric fields. In a crossbar configuration, these capacitors can perform neural network operations, especially MVM using simultaneous activations of word lines. As mentioned previously, their low-voltage operation (1.2 V) and low process temperature allow for smooth incorporation into 3-D stacked MVM units with standard CMOS circuits.

Figure 5a illustrates the IMC crossbar architecture with FeCaps as memory elements. Each column in crossbars employs an operational amplifier (OPAMP)-based charge summing amplifier to generate an analog value ($V_{out}$). This output $V_{out}$ reflects the result of the multiply-accumulate (MAC) operation, with the activations serving as inputs and the capacitance states of FeCaps acting as neural network weights. Hence, the generated MAC value, a summation of input-weight products, is a key operation for diverse ML workloads.

In the charge-based MAC computation process, two phases are involved. During the phase $\phi _1$, the bit lines ($BL_1$, $BL_2$, ..., $BL_N$) are set to the common mode voltage ($V_{CM}$), while the corresponding wordlines ($WL_1$, $WL_2$, ..., $WL_N$) are activated simultaneously to establish charges across the capacitors (pre-programmed to either HCS or LCS). In phase $\phi _2$, the word lines (WLs) are connected to $V_{CM}$, and the bit lines (BLs) to the summing capacitor ($C_{ref}$) which connects the input and output terminals of the OPAMP. This inference operation in crossbars is expressed by Eq. 1.

1 $$\begin{aligned} V_{out_j} = \frac{\sum \nolimits _{i=1}^N{(C_{FE_i} \times WL_i)}}{C_{ref}} \end{aligned}$$

Equation (1) encapsulates the charge accumulation process, where stored charges in the capacitors ($C_{FE_{i}}$) accumulate over $C_{ref}$. It also highlights the multiplication operation between input signals (activations, $WL_{i}$) and ferroelectric capacitance (weights/parameters, $C_{FE_{i}}$) in the MAC operation. Thus, the numerical values of the realized capacitance (HCS or LCS) represent the stored digital bit in the crossbar array. Figure 5b illustrates the analog MAC output voltage as a function of the number of activated WLs. In this scenario, all the FeCaps in a bitline *j* are programmed to an HCS. When the WLs are activated sequentially, the analog MAC voltage produced increases linearly with the number of WLs activated. The obtained result depicted in Fig. 5b is in line with previous studies that emphasize the substantial potential of FeCap crossbar arrays. Moreover, our analysis distinctively reveals that this instance shown in Fig. 5b and Ref.^18,19^ is realized under the most optimal condition for FeCap crossbar operation.

##### Challenges

Figure 5c presenting the timing diagram of a $4\times 4$ FeCap array illustrates an error occurrence during MAC operation. The diagram shows that when input activations are high (1111), and all weights are in LCS states (0000), the resulting accumulated output voltage is unexpectedly higher than in the scenario where the inputs and weights are set to an alternating pattern (1010). This indicates an erroneous output where the voltage corresponding to a digital ‘0’ (V$_x$) is greater than that for a digital ‘2’ (V$_y$). In other words, there is a random input-output correlation. This phenomenon presents a significant computational challenge and can lead to a reduction in the accuracy at the application level for DL tasks.

Our work has led to the important finding that computational errors are prevalent in many scenarios. The MAC output result, observed in Fig. 5b and in the previous works^18–20^, is the only specific condition when the FeCap crossbar can operate linearly. However, such conditions are not representations of practical applications. These errors are mainly connected to the low $C_{ratio}$ of FeCaps. Based on our experiment and other HZO-based metal-ferroelectric-metal (MFM) structure capacitors, $C_{ratio}$ falls within the range of 1.1–1.4. Such a small $C_{ratio}$ makes it difficult to discriminate their low and high capacitance states while activating multiple cells simultaneously. However, achieving a high $C_{ratio}$ is particularly challenging in MFM structure FeCaps due to inherent limitations associated with the properties of ferroelectric materials. This is because the varying dielectric constants of the FeCaps solely depend on the atomic-level dispersion between the atomic centers of positive and negative ions in ferroelectric materials.

**Figure 6:**
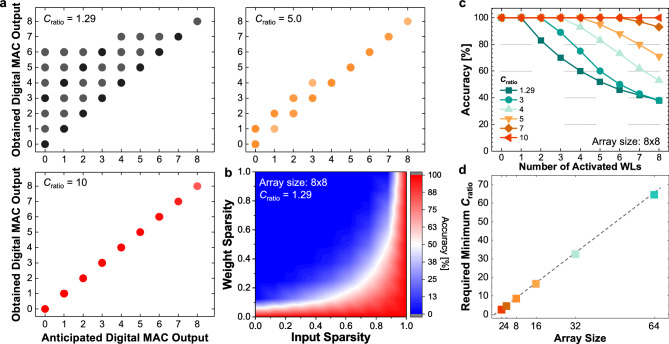
$8\times 8$ size FeCap crossbar array, operation schemes, and its performance. (**a**) Comparison of anticipated digital MAC output with obtained digital output from FeCap-based crossbars at $C_{ratio}$ = 1.29, 5.0, and 10. Cases with low $C_{ratio}$ exhibit considerable error in obtained MAC output originated from leakages during the activation of LCS. (**b**) Heatmap of accuracy with respect to input and weight sparsities at 1.29 of $C_{ratio}$. (**c**) Accuracy of MAC output for different $C_{ratio}$ and increasing number of activated WLs. (**d**) Minimum value of $C_{ratio}$ required for varying FeCap-based crossbar array sizes.

**Figure 7:**
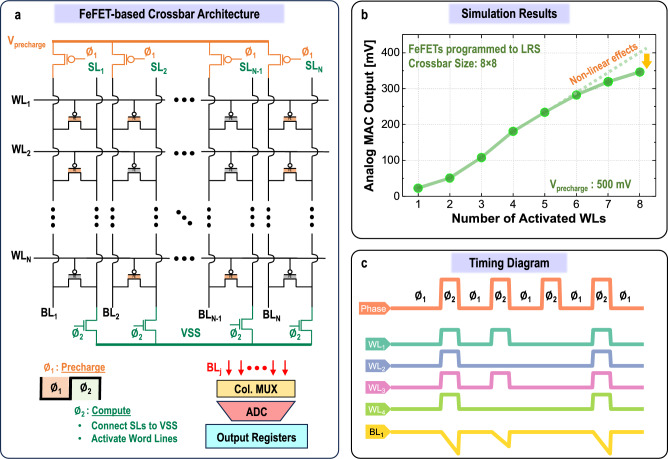
FeFET-based MAC operation. (**a**) Crossbar architecture with FeFETs as memory elements, tailored for IMC applications, utilizes a charge-based computational approach to execute MVM operations efficiently. (**b**) Simulation results for an $8\times 8$ crossbar array, showcasing variable input activation levels, with all FeFETs programmed to a state of 1. (**c**) Timing diagram illustrating the operation of a $4\times 4$ array, where all input elements are activated simultaneously.

Figure 6a illustrates the expected and obtained digital MAC outcomes for varying $C_{ratio}$ for a $8\times 8$ crossbar array, including $C_{ratio}$ values of 1.29 (the highest achievable from our experimental data), 5, and 10. Each instance (output) is derived from every 8-bit input and 8-bit weight sparsity pattern (i.e., 65,536 samples). These simulations support our claim that there is no direct correlation between the obtained and ideal digital MAC outputs at practical $C_{ratio}$ value of 1.29. We also revealed that the sparsity of the input and weight influences the output MAC values. Here, sparsity quantifies the percentage ratio of zero-valued elements to the total element count. Specifically, the more cases with a higher incidence of ‘1’ s in the inputs (denoting lower input sparsity) combined with ‘0’ s in the weights (denoting higher weight sparsity) are the more susceptible to errors. This finding is another cornerstone of our research. Figure 6b provides evidence that lower input sparsity and higher weight sparsity lead to an increased error. The low accuracy region corresponds to cases with higher weight sparsity (i.e., more LCS cells), while the red-colored area of higher accuracy correlates with scenarios featuring low weight sparsity and/or low input sparsity. Figure 6c presents the overall accuracy for output MAC values with varying numbers of input activations for different values of $C_{ratio}$ in a $8\times 8$ crossbar. As the number of activated WLs increases, accuracy drops quickly for lower $C_{ratio}$. This shows that a higher $C_{ratio}$ is correlated with a reduced probability of errors, with a $C_{ratio}$ of 10 for an $8\times 8$ crossbar array ensuring error-free operations. Figure 6d depicts the optimal $C_{ratio}$ for error-free operations in different array sizes. This analysis further underscores that larger arrays require higher $C_{ratio}$.

In array operations, ensuring disturb-free operation is important for the feasibility of FeCap, especially in selector-less cells. Despite the possibility of a small voltage read for non-destructive read^30^, there remains a risk that multiple read operations could lead to the destruction of the polarization states in FeCaps. This risk depends on various factors such as read conditions and device characteristics. Importantly, write operations are more vulnerable to disturbances compared to reading^31^. To alleviate this issue, several approaches such as utilizing recovering pulses, careful device development, and optimizing the write voltage could be a viable option.

#### FeFET crossbar arrays for IMC

Our fabricated FeFET device showed an off/on resistance ratio exceeding $10^4$, a leakage current below $10^{-12}$ amperes, and 1-V memory window, presenting FeFETs as an alternative for memory devices to ensure effective inference. Furthermore, like FeCaps, they can be accessed without the need for selectors, which contributes to improved memory density.

Previous studies mainly employed a current-based sensing scheme in FeFET crossbar arrays^32,33^. In this approach, the current drawn by each FeFET is integrated across a resistor, leading to an output voltage given by ($V_{out}= I_{total}\times R$). This summation is facilitated by a current-summing TIA in each column of the crossbar, a design reminiscent of the FeCap architecture as illustrated in Fig. 5a. While TIA is a necessity for a current-based sensing scheme, it introduces energy-intensive active circuitry, inevitably escalating the power demands, and reducing overall efficiency. Recent studies aimed to circumvent the need for a TIA by integrating current across the load capacitor to accumulate voltage^34,35^. However, this approach requires an additional capacitor in each column of the crossbar and requires large enough capacitance to counter wire parasitics, ultimately increasing chip footprint.

In this work, we proposed a charge-based approach in FeFET crossbars. This offers an alternative to TIA for current-to-voltage conversion and eliminates the need for extra bulky and area-intensive capacitors, achieving an energy-efficient and area-efficient design. Figure 7a provides an overview of the FeFET IMC crossbar and other auxiliary computing elements. The proposed method occurs in two phases. During the first phase $\phi _1$, the BLs are precharged to a potential denoted as $V_{precharge}$. In phase $\phi _2$, the WLs representing the input activations are enabled simultaneously. At the same time, the source lines (SLs) are connected to a lower potential (VSS). This configuration allows the BLs to discharge linearly based on the stored data (programmed FeFET state) and input activations. Consequently, this arrangement facilitates a MAC operation through integration along the BL. In this crossbar array, the $V_{precharge}$ applied to the BL and the voltage on the SL are crucial factors in regulating the current through the FeFETs during readout and directly influencing the discharge rate of the BLs. Additionally, careful sizing of the precharging PMOS transistor is required to ensure that the maximum current flow through the BLs is not constrained by the precharging circuit.

Figure 7b illustrates the simulated output MAC values for an $8 \times 8$ crossbar array. This setup is similar to the one used in Fig. 5b, where the WLs are sequentially activated while the FeFETs are programmed to 1 (high). Note that the issues observed in FeCaps crossbars (Fig. 6) do not apply here due to the high on-off ratio. The accompanying timing diagram in Fig. 7c, designed for a $4\times 4$ array for simplicity, elucidates the operational principle of the FeFET-based crossbar array.

##### Challenges

The implementation of FeFETs, despite their impressive on-off ratio characteristics, comes with the challenge of demanding higher programming operating voltages, typically in the range of 3–5 V. This necessitates the integration of separate charge-pumping circuits, especially as supply voltages continue to decrease in line with advancements in technology nodes. Moreover, unlike highly reliable FeCaps, FeFETs endurance on Si channels experience degraded endurance performance. This limits the number of training cycles, which prohibits on-chip learning.

In FeFET crossbars, increasing the number of parallel WL activations exacerbates non-idealities, as shown in Fig. 7b. These non-idealities arise from the non-linear *I-V* characteristics of FeFET, due to large discharge currents and reduced BL voltage. Such non-linearity becomes evident when activating multiple rows containing FeFETs programmed to ’1’ simultaneously with WLs ’1’ activations. Limiting the current helps avoid this issue, but reduces the resolution between subsequent output MAC levels, thereby imposing more precise performance for the subsequent peripheral sensing circuit.

### Opportunities for FeCaps and FeFETs in IMC

#### FeCaps

In pursuit of mitigating the $C_{ratio}$ constraints inherent to FeCaps, an effort has been devoted to exploring asymmetric structural configurations. One study employed different top and bottom electrode materials, achieving a memory window of $8.0 \times \epsilon _{0}$, with a maximum $C_{ratio}$ below 1.35^36^. While these efforts hold potential, our analysis (Fig. 6d) suggests that they may not yet meet the requirement for effective use in IMC. Recently, nano-laminate or superlattice structures have emerged as compelling means for enhancing the dielectric constant of devices^37–39^. These structures could offer the potential to mitigate the $C_{ratio}$ constraints by increasing the sensing margin. Another approach incorporated a semiconductor layer to draw extra charges (*dQ*), akin to FeFETs. This resulted in relatively high $C_{ratio}$ of 2.0^19^, 25^40^, and 125^41^. However, this approach, while achieving higher $C_{ratio}$s, demands notably higher programming voltages, such as 20 V^19^, 3.5 V^40^, and 6.5 V^41^, respectively. Additionally, it exhibits limited endurance compared to FeCaps^41^. Hence, there remains a compelling need for further exploration to push the boundaries of $C_{ratio}$ of MFM type FeCaps while maintaining low voltage operation and superior endurance characteristics. Beyond device engineering considerations, our research reveals that larger FeCap crossbar arrays tend to accumulate more errors compared to their smaller counterparts, as illustrated in Fig. S4. This observation may be attributed to the higher number of possibilities for the presence of LCS in larger arrays, emphasizing the impact of array size and weight sparsity on error accumulation. However, it is essential to note that this reduction in array size also comes at the cost of increased operation latency.

##### Approach 1: Pre-training with non-idealities of the existing FeCap crossbar array

Recognizing the constraints of FeCaps in achieving a larger $C_{ratio}$ and errors in IMC crossbars, our work aims to harness the superior characteristics of FeCaps, low-voltage operation, and enhanced reliability. To this end, we propose a pretraining approach designed to effectively train the crossbar architecture. This method strategically maps the data to the crossbar preemptively minimizing error-inducing scenarios, reducing the overall impact of LCS cells to a significant extent at the time of inference. By doing so, we substantially reduce the inaccuracies posed by LCS states, enabling the integration of FeCaps into IMC applications feasible.

**Figure 8:**
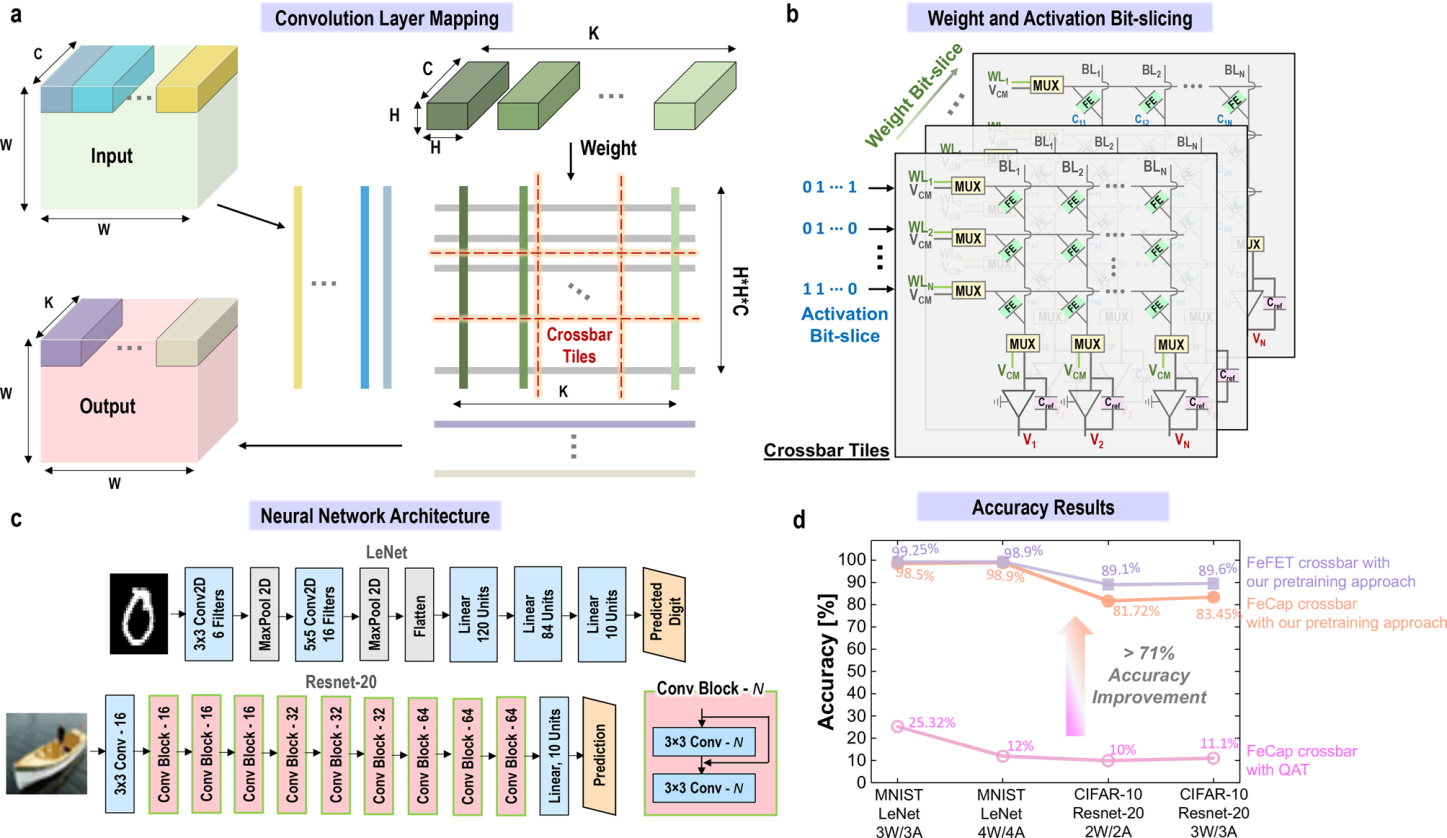
Pretraining approach for FeCaps-based neural network. (**a**) Convolution layer mapping to FeCap crossbars using Im2Col transform. (**b**) Demonstration of bit-slicing on input activations and stored weights for FeCap-based crossbars. (**c**) Neural network architectural layout of LeNet and Resnet-20 models. (**d**) Simulation results on MNIST and CIFAR-10 datasets for LeNet and Resnet-20 models comprised of FeCap and FeFET crossbars. FeCap crossbars based on Quantization-Aware Training (QAT) indicate lower accuracy. Accuracy evaluation of FeCap crossbars using our pretraining approach demonstrates significant accuracy improvement in FeCap-based neural network, comparable to the FeFET baseline. Note that QAT represents the training approach that only trains for quantization without considering the leakage of FeCaps.

This training approach, motivated by our previous works^42,43^, maps the neural network model to FeCap crossbars and trains the workload with weight, activation and partial sum quantization. Figure 8a shows a representative mapping of a convolution layer to crossbars, which involves converting the convolution operation into an MVM operation via Im2Col transform. Further, bit-slicing^44^ is introduced to extend the scalability of the training approach to accommodate multi-bit weight and activation precision. Quantized *N*-bit weight values are divided into *N* 1-bit bit-slices and stored in different FeCap crossbars, while quantized activations are bit-sliced to 1-bit values and applied on the crossbar across multiple compute cycles as shown in Fig. 8b. In our case, we consider $C_{ratio}$ of FeCaps to be 1.29, which means that ‘0’ weight values once mapped to the crossbar are represented as ‘0.77’ to emulate the LCS states introduced by the small $C_{ratio}$ of FeCaps. We compare the accuracy numbers obtained with two baselines: (1) a Quantization-Aware Training (QAT) approach which only trains for quantization without considering leakage of FeCaps, and (2) a FeFET baseline with near-ideal on/off characteristics trained with ‘0’ and ‘1’ weight only. The training approach is validated on commonly used computer vision datasets, MNIST, and CIFAR-10 datasets for different weight (W) and activation (A) precisions (Fig. 8d). Our observations indicate that the QAT approach, which is oblivious to low $C_{ratio}$ in FeCaps, achieves a catastrophically low accuracy, making the ML model unusable in practical scenarios. However, with our pretraining approach, we achieve accuracy approaching the FeFET baseline, benefiting from its high on-off ratio that effectively distinguishes between ‘0’ and ‘1’. For a smaller-scale MNIST dataset, we achieve accuracy within 1% of the ideal FeFET baseline. On the more challenging CIFAR-10 dataset, the accuracy degradation is 6.1% compared to the FeFET baseline with 3 bits of weight and activation precision.

**Figure 9:**
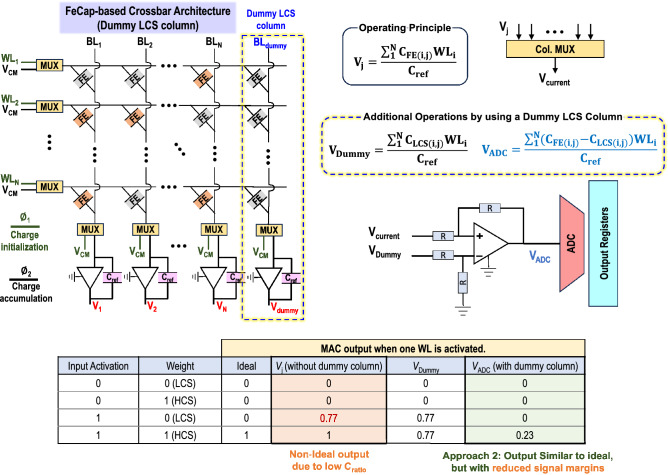
Approach 2, modified architecture for FeCap crossbars, incorporating a dummy column of LCS. The dummy column serves to subtract the leakage effects when activating LCS cells before digitizing. The table illustrates the different MAC outputs obtained by different architectures, highlighting the challenges of the reported architecture and demonstrating the effectiveness of the architecture in Approach 2.

##### Approach 2: Modified architecture with a dummy column of LCS

Including an additional dummy column of FeCaps, programmed for LCS, can also effectively mitigate the impact of leakage when activating LCS cells, a prevalent issue discussed in detail in earlier sections. Figure 9 illustrates the modified architecture with the dummy column. The dummy column operates in parallel with the other columns in the crossbars, sharing the WLs and input activations. Subsequently, the output of the dummy column undergoes subtraction from the output of each column of the crossbar before being fed to the ADC for digitization and further processing. To obtain the required MAC output, the OPAMP-based subtraction circuit is required as depicted in Eq. 2.

2 $$\begin{aligned} V_{out_j} = \frac{\sum \nolimits _{i=1}^N{([C_{FE_i}-C_{LCS_{dummy}}] \times WL_i)}}{C_{ref}} \end{aligned}$$

This architectural modification effectively eliminates the issues of leakage observed in previous works, leading to higher accuracy in computations (similar accuracy as with the FeFET case, shown in Fig. 8). However, it is essential to note that this method introduces additional TIA and subtractor circuits, thereby increasing design complexities, as well as the power and area requirements. For example, in a $8\times 8$ crossbar array, the architecture requires approximately $25\%$ higher power due to the TIAs, which consume significant power. Note that this overhead is reduced for larger crossbar arrays. Another important implication is the reduction of the signal margin due to subtraction, as depicted in the table shown in Fig. 9. This reduction may limit subsequent sensing circuits to operate on reduced margins, potentially extending the power budget.

#### FeFETs

The challenges of FeFETs are high-voltage operation and degraded endurance characteristics, which relate to energy efficiency and training capability. Notably, our observation of the FeCap structure (Fig. 2a) suggests that the operational voltage of FeFETs can potentially be mitigated by scaling the HZO thickness with an IL. Although this approach holds promise, it necessitates careful consideration of interfacial conditions between the Si channel and HZO layer or 1-nm IL layer. Despite the requirement for high voltage, which is applied to the gate electrode, FeFETs generally feature HZO thickness exceeding 4 nm. Consequently, the gate current drawn by high voltage remains significantly low (Fig. 4c). This exerts a negligible impact on the overall energy consumption of the system; however, additional circuit units are required to generate such high voltages. Furthermore, we made an effort on energy-efficient FeFET crossbar array by eliminating power-hungry and area-consuming components, i.e., TIAs and load capacitors. Our simulations show that a single OPAMP used as a TIA consumes 200 $\upmu$W of power (with a load cap of 200 fF and an operating frequency of 20 MHz). In contrast, our proposed charge-based scheme consumes approximately 10 $\mu$W of power for each column of an $8\times 8$ array. This power consumption is orders of magnitude lower than that of the current-based sensing scheme when applied to a complete $8\times 8$ array, which would necessitate eight such TIAs for iso-latency operations. Additional details on the TIA design, along with its power characteristics, are included in the supplementary information (Figs. S5 and S6).

The degraded reliability of FeFETs can be improved through several approaches. One method is to insert a high-dielectric constant material between the silicon channel and the HZO layer. This helps reduce the electric field across their interface, enhancing reliability^45^. Another approach involves substituting the channel material with alternatives like oxide semiconductors that can form cleaner interfaces with the HZO layer, potentially improving reliability^46^. Optimizing the programming pulses represents a promising approach to enhance the number of training iterations available for FeFETs. The endurance of ferroelectric devices is closely related to the electric field and its frequency during programming cycling, with shorter stress times leading to improved device endurance. To extend the potential for additional training cycles, it may be beneficial to employ a smaller electric field or shorter training pulse widths, thus increasing the reliability and longevity of FeFETs. Researchers have also put forward an off-chip/offline training approach^47,48^. This approach pertains to a situation in which a particular software is responsible for computing the optimal weight values for the DNN in a server during the training phase. These computed weights are subsequently transferred into the hardware for its subsequent inference processes to reduce the number of write-read-erase cycles. Similar strategies could also be applied to enhance the performance of FeFETs.

From a circuit perspective, activating a larger number of cells storing 1’s can drastically lower BL voltages, introducing nonidealities due to the non-linear characteristics of FeFETs. To mitigate this issue, sparsity of input activations and stored weights or limiting the discharge current through FeFETs can significantly minimize non-linearity in the analog MAC output. Notably, IMC architectures often employ tiling and bit-slicing techniques for both input activations and stored weights, thereby enabling MVMs to be performed in a bit-serial manner^49^. This bit-serial approach naturally introduces additional sparsity, increasing the proportion of zeros in both weights and inputs. Specifically, inputs are streamed in a bit-serial manner, while weights are sliced into bits and stored in the array cells. This arrangement is key for realizing high-precision MVMs, as elaborated in Fig. 8b. For example, such weight slicing can yield a bit-level sparsity of at least 60%^50^. This increased sparsity helps activate a greater number of rows during MAC operations and reduces the occurrence of sensing inaccuracies by the nonidealities.

## Discussion

In this study, we investigated the capabilities of FeCaps and FeFETs for executing ML workloads using IMC. Both FeCaps and FeFETs demonstrate robust selector-less operation while providing high internal resistance compared to wire resistances in crossbars. This mitigates the effects of IR drop and sneak paths, improving area and energy efficiency. At the device level, strategic innovations were realized through the integration of an IL with thin HZO for low-voltage operation, low-temperature process, and improved reliability of FeCaps. This integration signifies an important step towards energy-efficient devices, compatible with CMOS technology voltage scaling.

A physics-based model is used to characterize our fabricated devices to evaluate their performance in IMC crossbars. To ensure accurate computations, it is essential to ensure high $C_{ratio}$. However, due to the inherent properties of ferroelectric materials, it is difficult to achieve a high $C_{ratio}$. Consequently, we encountered a degradation in accuracy in the FeCap crossbars. To address this problem, we propose two approaches: (i) a system-algorithm co-design solution, which considers the effect of inherent low $C_{ratio}$ of FeCaps during the training phase of the neural network, resulting in an improvement in accuracy from 10.0% to 81.7% for the CIFAR-10 dataset, and (ii) an architectural solution, which introduces an additional dummy column that subtracts the leakage effect from active columns before digitizing. However, this solution comes at the cost of increased area and power budget.

On the contrary, FeFETs benefit from their high on-off ratio, leveraging the superior switching properties of transistors. Our analysis indicates that FeFETs outperform FeCaps in IMC crossbars and achieve higher network-level accuracy (89.1% on CIFAR-10). However, they demand higher programming voltages and compromised reliability compared to FeCaps. Note that these higher programming voltages do not significantly impact overall energy consumption, but may necessitate additional interfacing circuitry, such as charge-pumping circuits. Also, our proposed charge-based computing scheme considerably reduces energy consumption in the FeFETs crossbar by eliminating power-intensive TIAs for current-to-voltage conversion and bulky capacitors for voltage accumulation as used in previous approaches. In conclusion, this work provides comprehensive exploration and analyses, enhancing our understanding of the potential and challenges of employing FeCaps and FeFETs in IMC for advanced ML applications.

## Methods

### FeCap fabrication

The device fabrication started with cleanings of *p*$^+$-doped Si substrate. The cleanings were composed of four stages: (1) piranha cleaning (H$_{2}\text {SO}_{4}$ : H$_{2}$O = 3: 1) at 120$^{\circ }$C for 10 min, (2) SC1 cleaning (NH$_{4}$OH : H$_{2}\text {O}_{2}$ : deionized (D.I.) water = 1 : 1 : (5) at 80 $^{\circ }$C for 10 min, (3) SC2 cleaning (HCl : H$_{2}\text {O}_{2}$ : D.I. water = 1:1: (6) at 80 $^{\circ }$C for 10 min, (4) DHF cleaning (HF:D.I. water = 1:100) at room temperature for 30 sec and extra few seconds more for completely sheet off the native oxide (made completely hydrophobic surface). Immediately following the cleaning, the samples were moved to N$_{2}$ glove box and Atomic Layer Deposition (ALD) depositions were processed. The 1-nm ALD IL materials of $\text {HfO}_{2}$, $\text {ZrO}_{2}$, and $\text {Al}_{2}\text {O}_{3}$ were deposited at 200 $^{\circ }$C, using [($\text {CH}_{3}$)$_{2}$N]$_{4}$Hf (TDMAHf), [($\text {CH}_{3}$)$_{2}$N]$_{4}$Zr (TDMAZr), ($\text {CH}_{3})_{3}$Al (TMA) and H$_{2}$ O for the precursors Hf, Zr, Al and O, respectively. The ALD $\text {Hf}_{0.5}\text {Zr}_{0.5}\text {O}_{2}$ (HZO) film was deposited by alternatively depositing one cycle of $\text {HfO}_{2}$ and one cycle of $\text {ZrO}_{2}$ at 200 $^{\circ }$C in the same chamber of ALD IL. After the deposition of HZO/IL dielectric stacks, the 20-nm ALD TiN is deposited for a capping layer. Subsequently, three cycles of toluene, acetone, and IPA cleanings were conducted right before the Rapid-Thermal Annealing process (RTA). The cleaned samples underwent a post-metal annealing process for 1 min in N$_{2}$ ambient by RTA. The temperatures for the Post-Metal Annealing (PMA) (T$_{PMA}$) were 275, 300, 350, 400, and 500 $^{\circ }$C. The photolithograph processes were conducted to form capacitor patterns. Then, 180 nm Al was deposited by e-beam evaporator and soaked in acetone solution for 12 hours for lift-off. For device isolation, dry etching was performed for TiN etching.

### FeFET Fabrication

On the silicon-on-insulator (SOI) wafer, initial cleanings (SC1, SC2, and DHF) and SOI layer thinning through dry oxidation to 35 nm were performed followed by channel doping. Active isolation and source/drain implantation were proceeded followed by RTA at 1000 $^\circ$C for 30 sec. Before HZO deposition, H$_{2}\text {O}_{2}$ cleaning for 90 sec was conducted to form a $\text {SiO}_{2}$ interfacial layer. Right after the cleaning process, 2-nm $\text {Al}_{2}\text {O}_{3}$/10-nm HZO/1-nm $\text {Al}_{2}\text {O}_{3}$ were serially deposited by ALD. The first $\text {Al}_{2}\text {O}_{3}$ layer effectively quenches Hf ions from diffusing into $\text {SiO}_{2}$ layer, which could result in soft phonon scattering. Thereafter, Ni was deposited for ohmic contacts, and the ferroelectricity activation and silicide were performed together by RTA at 500 $^\circ$C for 30 s. Metal gate and contact pads were formed followed by forming gas annealing.

### Device measurement and characterization

*P*-*E* measurement, endurance, and retention were carried out with a Radiant RT66C with an aid of pulse generator Agilent 33220A. *J*-*E* measurement was done with a Keysight B1500A. For the *C*-*E* measurement, an Agilent E4980A LCR meter was used.

### Circuit simulation methodology

The entire simulation is performed in a commercial SPICE simulator, Cadence Spectre, which includes analyzing the device characterization and crossbar analysis for Read/Write and MVM operations with ferroelectric devices. These ferroelectric devices are modeled using a Presiach-based model, which is fitted to our experimental/measurement results from our fabricated devices. We analyzed the polarization and realized capacitance values in different scenarios to fit the Presiach model to the measurement data. To support high-voltage operation for FeFETs, we have used an I/O transistor from the TSMC 65nm technology library, which is connected to the FeCap model to form the FeFET cell, which is the building block for all FeFET-based crossbar array simulations, while the standalone FeCap model is used for FeCap-based simulations. Other auxiliary blocks, like amplifiers, PMOS precharging blocks, etc. also are built in the 65-nm technology nodes. To perform an extensive crossbar analysis, we have coded a script in Python to generate different patterns of input and weight sparsity for varying $C_{ratio}$. The values of $C_{ratio}$ and capacitance at LCS used in the Python script are first set to values obtained from fabricated devices to ensure proper replication of results from SPICE simulations.

### Neural network training methodology

The training of neural networks on CIFAR-10 and MNIST is performed in Pytorch. For incorporating crossbar specific computations including Im2Col operation and bit-slicing, we implemented a custom implementation of convolution and linear layers in Pytorch. The weight and activation quantization functions are adopted from LSQ^51^. The LeNet model is trained on the MNIST dataset for 100 epochs, while the ResNet-20 model is trained on CIFAR-10 for 200 epochs. For both models, we use an initial learning rate of 0.1 and use cosine annealing learning rate scheduler. The models are trained using a Stochastic Gradient Descent (SGD) optimizer^52^ with a momentum of 0.9 and weight decay of $10^{-4}$.

## Supplementary Information

Supplementary Information.

## Data Availability

The datasets used and/or analysed during the current study available from the corresponding author on reasonable request.
