# Supplementary material for: Ferroelectric capacitors and field-effect transistors as in-memory computing elements for machine learning workloads
Source: Sci Rep. 2024 Apr 24;14:9426. doi: 10.1038/s41598-024-59298-8 (PMC11551200; doi:10.1038/s41598-024-59298-8)
Supplement: Supplementary file 1 — Supplementary Information. [file 41598_2024_59298_MOESM1_ESM.pdf]

Supplementary Information for

Ferroelectric Capacitors and Field-Effect Transistors as In-Memory Computing Elements for Machine Learning Workloads

Eunseon Yu[1,+], Gaurav Kumar K[1,+], Utkarsh Saxena[1], and Kaushik Roy[1,*]

[1]School of Electrical and Computer Engineering, Purdue University, West Lafayette, IN 47907, USA.

*Address correspondence to:

kaushik@purdue.edu (K. Roy)

[+]contributed equally to this work

Table of Contents

Section I. Ferroelectric Device Measurement

Section II. Crossbar Array Analysis

Section III. Charge-based and Current-based Inference Circuits and their Comparison

- **i. Design of Operational Amplifier (OPAMP)**
- **ii. Simulated Results for OPAMP**

Section IV. Neural Network Training Details

- **i. Datasets**
- **ii. Neural Network Models**
- **iii. Weight and Activations Quantization**

Section I. Ferroelectric Device Measurement

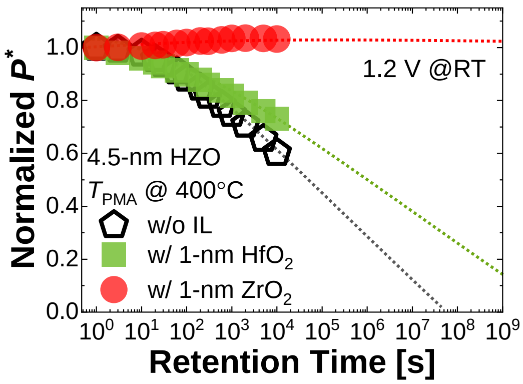

Fig. S1. Retention time measurement after programming operation at 1.2 V.

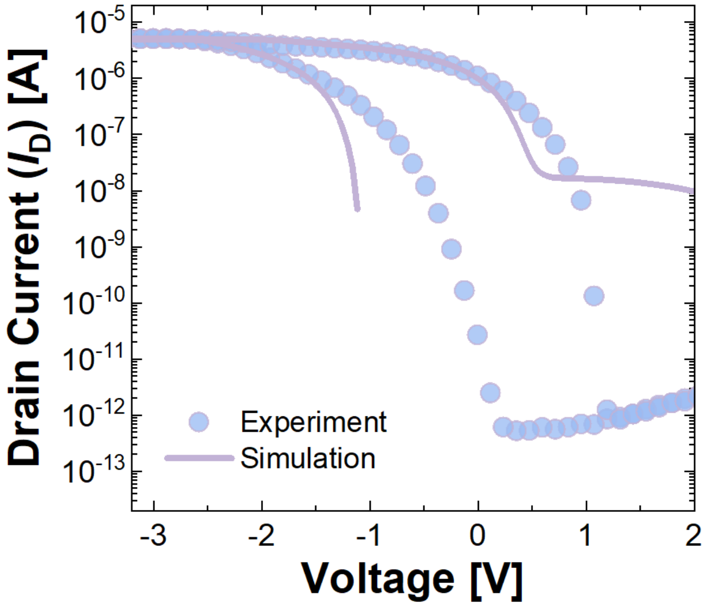

Fig. S2. Semi-log scale transfer characteristics of our fabricated *p*-type FeFET with simulation model. Although fitting the leakage current was challenging, our fitting meets the saturation current and the threshold voltage to our fabrication and this disparity does not compromise the validity of our crossbar analyses for Matrix-Vector Multiplication (MVM) computations. The saturated drain current at around 5×10^{-6} A is because of the high source and drain contact resistances.

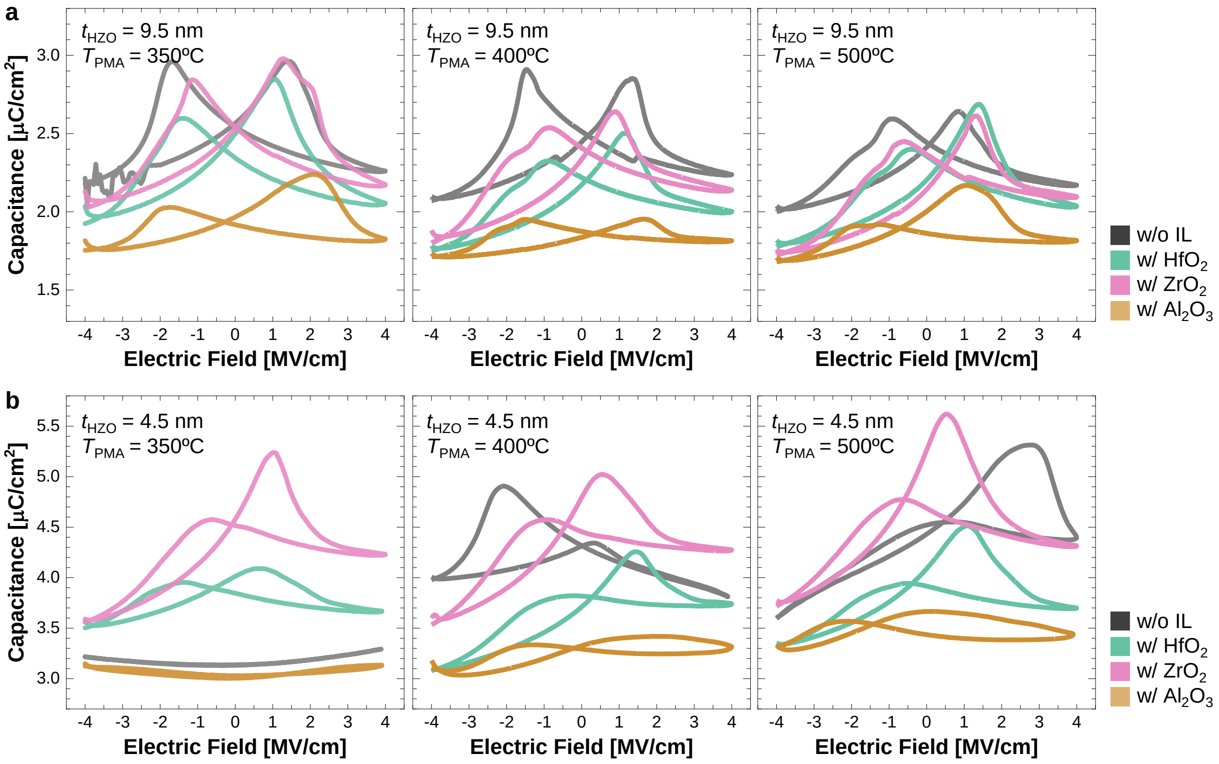

Fig. S3. Capacitance–electric field (*C*-*E*) characteristics for different interfacial layer materials, different post-metal annealing temperature (T_{PMA}) with **a** 9.5 nm HZO and **b** 4.5 nm HZO. Small signal frequency was fixed to 1 kHz.

Section II. Crossbar Array Analysis

FeCap crossbar arrays are simulated at the different input and weight sparsity cases. In this regard, different crossbars of sizes 8×8, 32×32, 64×64, and 128×128 are simulated with ADC precision of 3, 5, 6, and 7 bits respectively. The colormap in Fig. S3 indicates multiply-and-accumulation (MAC) operation accuracy which is calculated for 10,000 randomly generated inputs and weights for different sparsity levels. Smaller crossbars perform better than larger ones in terms of MAC operation accuracy, as can be seen from the red regions. This can be mainly attributed to the smaller possible scenarios of the presence of LCS in weights. Note MAC operation accuracy, which is the accuracy of the performed MAC operation when all the inputs are activated parallelly for all rows of the weights, does not represent neural network accuracy but fundamentally contributes to it.

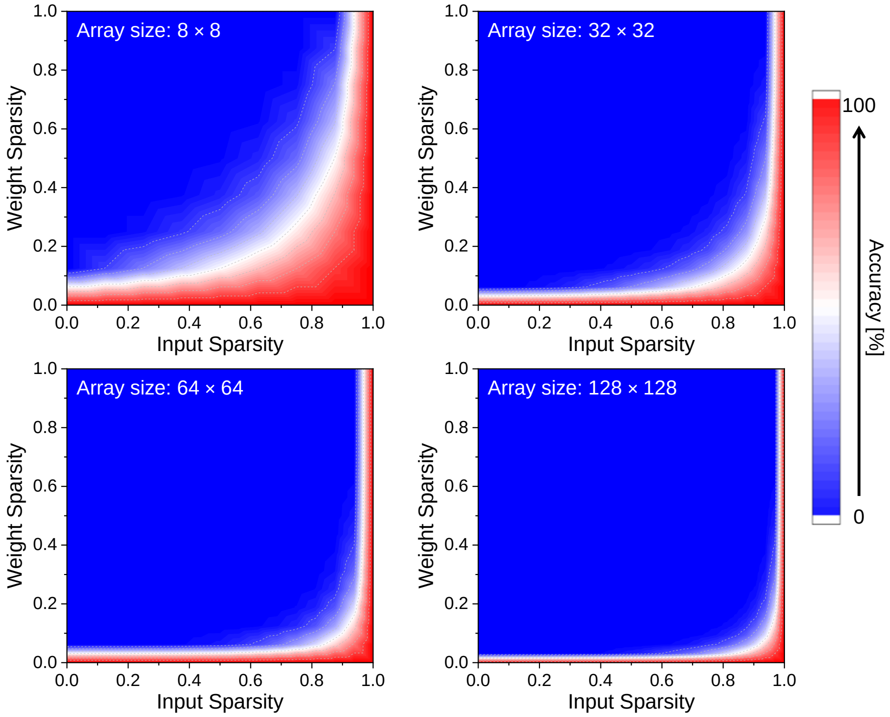

Fig. S4. Accuracy of MAC operation with FeCaps having 1.29 of C_{ratio}. Simulated capacitive array sizes are 8×8, 32×32, 64×64. and 128×128.

Section III. Charge-based and Current-based Inference Circuits using OPAMPs

i. Design of Operational Amplifier (OPAMP)

The operational amplifier (OPAMP) is a crucial component in electronic circuits, serving as a differential voltage amplifier with high input impedance and low output impedance. Its main purpose is to amplify the voltage difference between two input terminals. The op-amp, configured in various ways, plays a vital role in signal processing, analog computation, and the design of diverse electronic circuits. In this work, we have utilized the op-amp as a trans-impedance amplifier (TIA) for analyzing current-based sensing techniques in FeFET crossbars and as a charge-summing amplifier for charge-based sensing in FeCAP crossbars.

The accompanying figure (Fig. S4) illustrates the circuit-level implementation of the op-amp. In our design, a PMOS input stage enables the low-power operation of the op-amp, providing a tunable input dynamic range to reduce the power budget. It also ensures linearity within the operating range. The design features a three-stage op-amp: the first two stages provide gain, and the last stage is a voltage follower to facilitate driving capabilities. The supply voltage to the op-amp can be adjusted to obtain more input range.

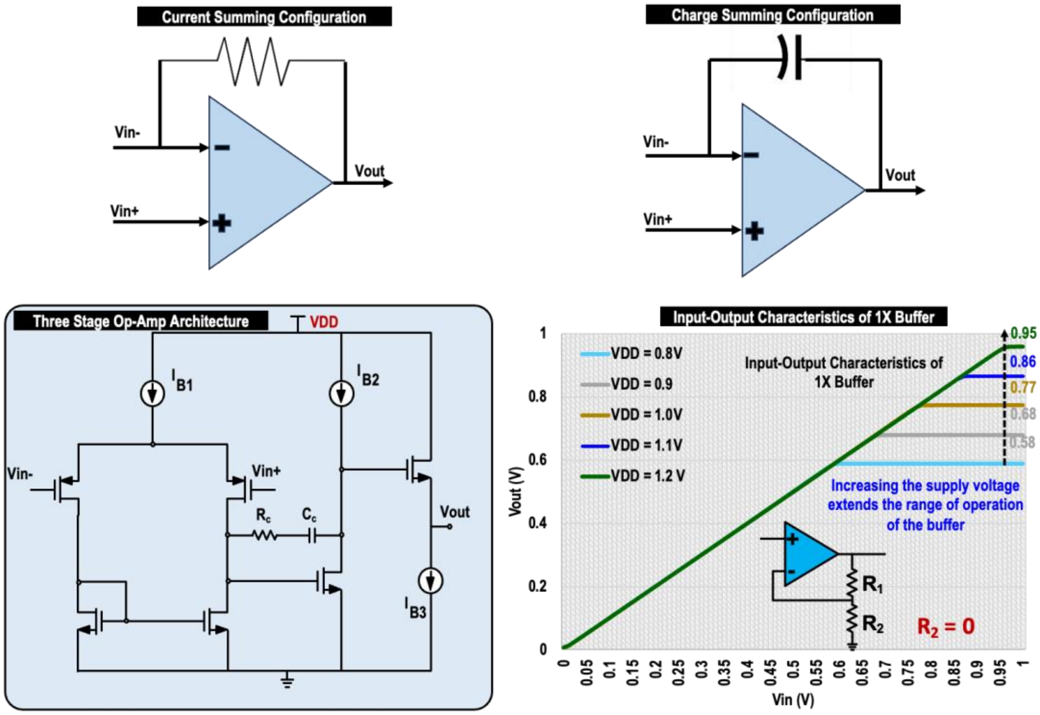

Fig. S5. Operational amplifier (OPAMP) configurations for charge and current summing employed in FeCap and FeFET crossbars for sensing. The three-stage architecture of the OPAMP is illustrated, along with the input-output characteristics for the buffer for varying supply voltages.

ii. Simulated Results for OPAMP

Figure S5 illustrates the power consumption characteristics of the OPAMP for varying load capacitances and operating frequencies. The graph depicts the power versus load capacitance maintaining a fixed operating frequency of 20 MHz while varying the loads accordingly. Conversely, the graph depicting power versus operating frequency holds the load capacitance constant at 200 fF, with variable frequency.

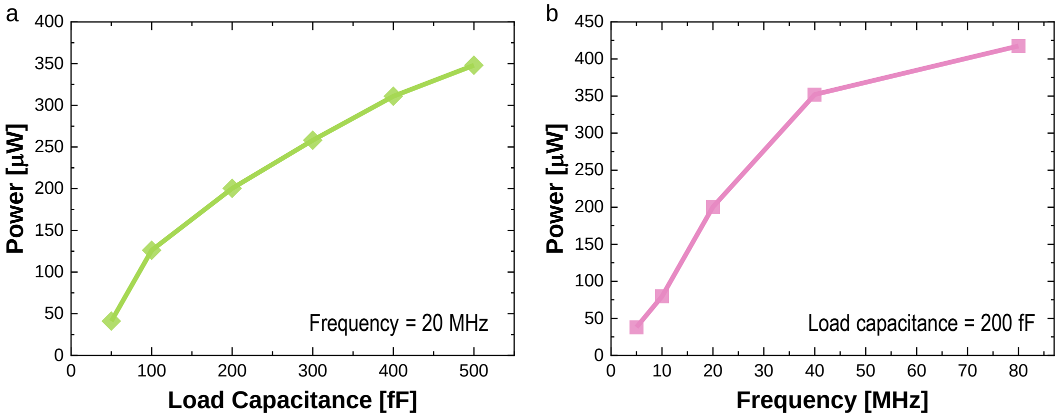

Fig. S6. Power consumption characteristics of the operational amplifier (OPAMP) are evaluated under varying load capacitance and operating frequency. Two separate analyses are presented: **a** Power vs Load Capacitance, where the operating frequency is fixed at 20 MHz, and **b** Power vs Operating Frequency, where the load capacitance is set to 200 fF.

Section IV. Neural Network Training Details

i. Datasets

- **MNIST**

MNIST, standing for Modified National Institute of Standards and Technology, is a renowned dataset frequently utilized in machine learning. It comprises 60,000 training images and 10,000 testing images, each being a 28×28 grayscale representation of handwritten digits (0 through 9). This dataset serves as a fundamental benchmark for assessing and developing image processing and classification algorithms, particularly in the realm of digit recognition. Its simplicity and effectiveness have positioned it as a cornerstone for introducing individuals to the intricacies of image-based machine learning tasks.

- **CIFAR-10**

CIFAR-10, derived from the Canadian Institute for Advanced Research, is a pivotal dataset in the domain of machine learning. It encompasses 60,000 color images (3 input channels), each measuring 32×32 pixels, distributed across 10 distinct classes. These classes range from airplanes and automobiles to birds, cats, and various other categories. CIFAR-10 serves as a robust benchmark for training and evaluating image classification algorithms. Its diverse set of images poses a challenge, making it a valuable resource for researchers and practitioners delving into the intricacies of computer vision.

ii. Neural Network Models

- **LeNet**

LeNet, designed by Yann LeCun and his collaborators, is a pioneering convolutional neural network (CNN) architecture. Proposed in the early 1990s, LeNet played a pivotal role in advancing the field of computer vision, particularly in handwritten digit recognition. The architecture consists of multiple layers, including convolutional layers, subsampling layers, and fully connected layers.

In its original form, LeNet comprises seven layers, with a unique structure that leverages convolutional and subsampling operations to capture spatial hierarchies within the input data. LeNet's design also incorporates activation functions, such as the hyperbolic tangent (tanh) and the sigmoid function, enhancing the model's non-linear learning capabilities.

LeNet has significantly influenced subsequent CNN architectures and laid the groundwork for modern deep learning applications in image recognition and classification tasks. While variations and adaptations have emerged over the years, LeNet remains a foundational model in the history of convolutional neural networks.

- **ResNet-20**

ResNet-20 is a convolutional neural network (CNN) architecture that falls under the Residual Network (ResNet) paradigm. Specifically, it consists of 20 layers, featuring a deep stack of convolutional and residual blocks. The key innovation in ResNet-20 is the introduction of residual connections, allowing the network to learn residual mappings. These connections facilitate the direct flow of information from one layer to another, mitigating the vanishing gradient problem during training of deep networks.

The residual blocks within ResNet-20 comprise multiple convolutional layers with batch normalization and rectified linear unit (ReLU) activations. These blocks enable the model to capture intricate hierarchical features from input data. ResNet-20 has demonstrated superior performance in image classification tasks by effectively leveraging the advantages of residual learning, making it a prominent architecture in the landscape of deep neural networks.

iii. Weight and Activation Quantization

The weight and activation quantization function are adopted from LSQ[S1]. It involves mapping the floating-point weight/activation values to the quantization grid with the help of a scaling factor. The scaling factors are different for layer-wise weight and activations. The quantization function is given below,

$v_q = s \cdot \left[clamp\left(\frac{v}{s}, Q_N, Q_P\right)\right]$, where Q_N and Q_P are the quantization bounds.

For n -bit signed weights, $Q_P = 2^{n-1} - 1$ and $Q_N = -2^{n-1}$. While for unsigned activations (obtained after ReLU) activation function, $Q_P = 2^n - 1$ and $Q_N = 0$.

References

[S1] Steven K. Esser; Jeffrey L. McKinstry; Deepika Bablani; Rathinakumar Appuswamy; Dharmendra S. Modha. Learned Step size Quantization. *International Conference on Learning Representation* **2020.**
